# Modification of a Single Atom Affects the Physical Properties of Double Fluorinated Fmoc-Phe Derivatives

**DOI:** 10.3390/ijms22179634

**Published:** 2021-09-06

**Authors:** Moran Aviv, Dana Cohen-Gerassi, Asuka A. Orr, Rajkumar Misra, Zohar A. Arnon, Linda J. W. Shimon, Yosi Shacham-Diamand, Phanourios Tamamis, Lihi Adler-Abramovich

**Affiliations:** 1 Department of Oral Biology, The Goldschleger School of Dental Medicine, Sackler Faculty of Medicine, Tel Aviv University, Tel Aviv 6997801, Israel; dr.moranaviv@gmail.com (M.A.); dana21cohen@gmail.com (D.C.-G.); rajkumarmisra96@gmail.com (R.M.); zoharnon@gmail.com (Z.A.A.); 2 The Center for Nanoscience and Nanotechnology, Tel Aviv University, Tel Aviv 6997801, Israel; 3 The Center for the Physics and Chemistry of Living Systems, Tel Aviv University, Tel Aviv 6997801, Israel; 4 School of Mechanical Engineering, Afeka Tel Aviv Academic College of Engineering, Tel Aviv 6910717, Israel; 5 Department of Materials Science and Engineering, Iby and Aladar Fleischman Faculty of Engineering, Tel Aviv University, Tel Aviv 6997801, Israel; yosish@tauex.tau.ac.il; 6 Artie McFerrin Department of Chemical Engineering, Texas A&M University, College Station, TX 77843-3122, USA; asukaorr@tamu.edu (A.A.O.); tamamis@tamu.edu (P.T.); 7 Department of Chemical Research Support, Weizmann Institute of Science, Rehovot 76132701, Israel; linda.Shimon@weizmann.ac.il; 8 Department of Physical Electronics, School of Electrical Engineering, Faculty of Engineering, Tel Aviv University, Tel Aviv 69978, Israel; 9 TAU/TiET Food Security Center of Excellence (T2FSCoE), Thapar Institute of Engineering and Technology, Patiala 147004, India; 10 Department of Materials Science and Engineering, Texas A&M University, College Station, TX 77843-3003, USA

**Keywords:** self-assembly, low-molecular-weight hydrogelator, phase-transition, molecular-dynamics

## Abstract

Supramolecular hydrogels formed by the self-assembly of amino-acid based gelators are receiving increasing attention from the fields of biomedicine and material science. Self-assembled systems exhibit well-ordered functional architectures and unique physicochemical properties. However, the control over the kinetics and mechanical properties of the end-products remains puzzling. A minimal alteration of the chemical environment could cause a significant impact. In this context, we report the effects of modifying the position of a single atom on the properties and kinetics of the self-assembly process. A combination of experimental and computational methods, used to investigate double-fluorinated Fmoc-Phe derivatives, Fmoc-3,4F-Phe and Fmoc-3,5F-Phe, reveals the unique effects of modifying the position of a single fluorine on the self-assembly process, and the physical properties of the product. The presence of significant physical and morphological differences between the two derivatives was verified by molecular-dynamics simulations. Analysis of the spontaneous phase-transition of both building blocks, as well as crystal X-ray diffraction to determine the molecular structure of Fmoc-3,4F-Phe, are in good agreement with known changes in the Phe fluorination pattern and highlight the effect of a single atom position on the self-assembly process. These findings prove that fluorination is an effective strategy to influence supramolecular organization on the nanoscale. Moreover, we believe that a deep understanding of the self-assembly process may provide fundamental insights that will facilitate the development of optimal amino-acid-based low-molecular-weight hydrogelators for a wide range of applications.

## 1. Introduction

Supramolecular self-assembly based on noncovalent interactions between monomeric building blocks is a powerful strategy for the design of well-ordered functional architectures [1,2,3,4]. Due to their unique mechanical and physicochemical properties, such assembled systems show great promise in a wide range of applications, including electro-optics [5], biomedicine [6,7], chemical separation [8], and material science [9]. These supramolecular assemblies form a rich variety of nano- and micro-architectures including tubes, fibers, films, plates, and vesicles [10,11,12]. 

Low-molecular-weight peptides and amino acid derivatives that self-assemble to form ordered nanostructures have attracted significant attention in recent years. Their natural ability to form supramolecular networks can be utilized for biomedical applications including controlled drug delivery [13], vaccine development [14], tissue engineering [15], and regenerative medicine [16]. The orderly nanostructures resulting from the self-assembly process contain large internal cavities, capable of trapping large quantities of water molecules, and allow these building blocks to serve as gelators for self-supporting hydrogels [17,18,19]. 

Since the early work of Janmey and co-workers who developed hydrogels based on Fmoc-Leu-Asp [20], and the later work of Xu and co-workers who developed nanofibrous hydrogels based on Fmoc-D-Ala-D-Ala [11], there has been a keen interest in fluorenylmethoxycarbonyl (Fmoc) protected amino acids and dipeptides and their possible applications [5,21,22]. The main advantages of these materials are their ease of synthesis, low cost, similarity to the natural extracellular matrix, and good biocompatibility. 

Among the molecular hydrogelators reported to date, those based on Fmoc-phenylalanine (Fmoc-Phe) have attracted particular interest due to the wide variety of unique properties that can readily be obtained by chemical and biological decoration. In addition, the assembly properties of Fmoc-Phe can be greatly enhanced by the incorporation of various substituents, including halogens, on the benzyl side chain [23,24,25]. Incorporation of single halogen substituents on the aromatic side chain of Fmoc-Phe was shown to enhance the efficient self-assembly of these amino acid derivatives dramatically (relative to Fmoc-Phe), and to produce hydrogel fibril networks that promote hydrogelation in aqueous solvents [23,24,25]. Other studies have reported that the position of halogen substitution (ortho, meta, para) and the halogen itself (F, Cl, Br) have a strong influence on the self-assembly and hydrogelation rate, and the emergent viscoelasticity of the resulting hydrogels [24,26,27,28]. These findings indicate that the transducer type and position have a profound influence on the molecular self-assembly process and the resulting properties. 

Another well-studied Fmoc-Phe derivative is Fmoc-pentafluoro-phenylalanine (Fmoc-F_5_-Phe) that was previously shown to form a hydrogel [25]. Recently, we demonstrated that Fmoc-F_5_-Phe undergoes a phase transition from spheres to a fibrillary gel, and ultimately to crystals [29]. When co-assembled with either Fmoc-F_5_-Phe-PEG or Fmoc-Phe-Phe, this building block exhibits ideal stress-responsive behavior [30] or synergistic improvement of the mechanical properties [31], respectively. Moreover, we recently demonstrated the antibacterial activity of Fmoc-F_5_-Phe against *Streptococcus mutans* in dental composite restoratives [32] and described its use as an antibacterial coating for different surfaces [33]. 

The present study reports the use of a combination of experimental and computational methods to investigate the molecular self-assembly process and phase transition during gelation of two previously unreported Fmoc-Phe derivatives: Fmoc-3,4-difluoro-phenylalanine (Fmoc-3,4F-Phe), and Fmoc-3,5-difluoro-phenylalanine (Fmoc-3,5F-Phe). In addition, we used X-ray diffraction (XRD) and molecular dynamics (MD) simulations to examine how changing the position of a single fluorine in the aromatic ring of Fmoc-Phe hydrogels, affects their physical properties, including self-assembly kinetics, morphology, physical characterization, phase transition as well as structural characterization at the atomic and molecular level. 

## 2. Results and Discussion

### 2.1. Kinetic Analysis and Morphology of Double-Fluorinated Fmoc-Phe Hydrogels 

**Figure 1:**
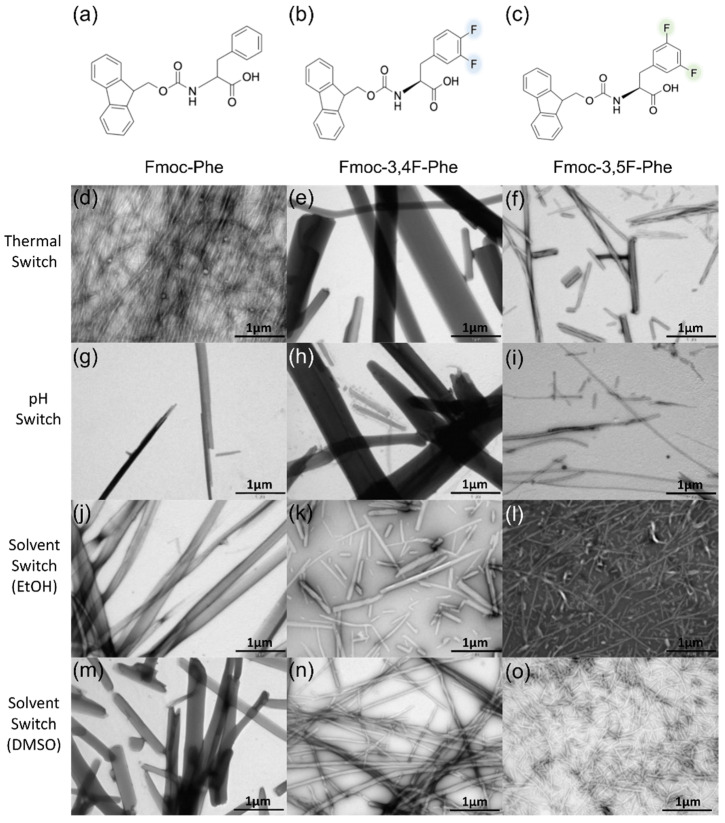
Formation of double fluorinated Fmoc-Phe hydrogels. Molecular structure of (**a**) Fmoc-Phe, (**b**) Fmoc-3,4F-Phe, and (**c**) Fmoc-3,5F-Phe. (**d**–**o**) TEM images of the structures formed by the various methods (**d**–**f**) Thermal Switch (**g**–**i**) pH Switch (**j**–**l**) Solvent Switch (EtOH) (**m**–**o**) Solvent Switch (DMSO), scale bar 1 µm.

Inspired by the self-assembly of Fmoc-Phe and the formation of hydrogel, we studied two new double fluorinated Fmoc-Phe building blocks: Fmoc-3,4F-Phe and Fmoc-3,5F-Phe (Figure 1a–c). Whereas Fmoc-3,4F-Phe consist of two adjacent fluorine atoms, the fluorine atoms in the Fmoc-3,5F-Phe are farther apart, resulting in a lower electron repulsion. We examined the propensity of the building block to form self-supporting 3D hydrogel in response to a thermal switch, pH switch, or solvent switch using ethanol (EtOH) and dimethyl sulfoxide (DMSO) (Figure S1, Supporting Information). 

In order to investigate the morphological structure and the alterations between the derivatives, we performed transmission electron microscopy (TEM) analysis (Figure 1). All the conditions tested exhibited fibrillary network morphology typical of supramolecular gels (Figure 1d–o). However, rapid formation of stable and rigid hydrogels (within a few minutes) was seen only when the solvent switch method, using DMSO as the organic solvent, was employed. For this reason, further comprehensive study of the Fmoc-Phe derivatives employed only this method. Comparing the nanostructures of the different hydrogels revealed that while Fmoc-Phe forms thick fibrils with an average diameter of 175 nm (Figure 1m), Fmoc-3,4F-Phe exhibits thinner and partly tangled fibrils, approximately 45 nm in diameter (Figure 1n), and the fibrils formed by Fmoc-3,5F-Phe were the thinnest with an average diameter of 30 nm (Figure 1o). 

**Figure 2:**
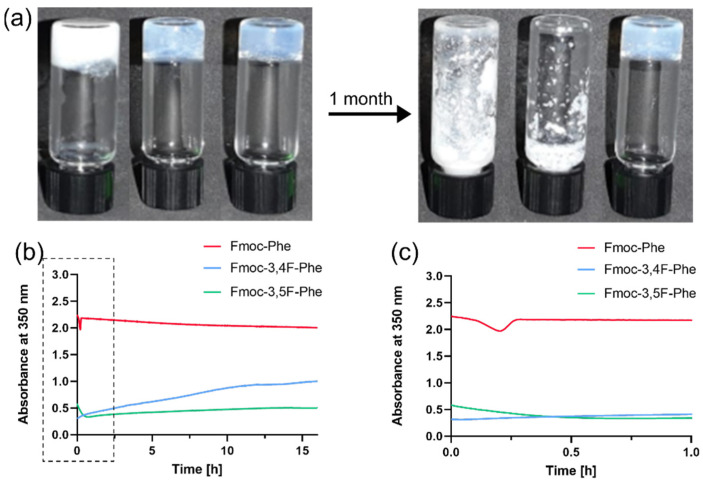
Kinetic characterization of the assemblies. (**a**) Hydrogels formed from Fmoc-Phe derivatives one-hour post gelation (left side) and one-month post gelation (right side). Vials from left to right: Fmoc-Phe, Fmoc-3,4F-Phe, and Fmoc-3,5F-Phe. (**b**) OD kinetics at 350 nm for the first 16 h of hydrogel formation. (**c**) Higher scale enlargement of the OD kinetics for the first hour (dashed area).

The gelation process of low-molecular-weight building blocks in the solvent switch method is usually characterized by an optical change from an opaque solution to a more transparent hydrogel [24,34] as organized nanostructures are formed [34]. While Fmoc-Phe formed an opaque hydrogel, both Fmoc-3,4F-Phe and Fmoc-3,5F-Phe formed transparent hydrogels (Figure 2a). Neither the Fmoc-Phe nor the Fmoc-3,4F-Phe hydrogel was stable, and phase separation was observed after one week, whereas the Fmoc-3,5F-Phe hydrogel was stable over time and maintained a clear 3D self-supporting hydrogel structure for at least 1 month. 

The kinetics of the self-assembly process to nanostructures was monitored by measuring the turbidity of the solution at a wavelength of 350 nm over time (Figure 2b). This revealed that the absorbance of Fmoc-Phe remained high and stable over 16 h with an optical density (OD) of more than 2. In contrast, the absorbances of the double-fluorinated Fmoc-Phe hydrogels were much lower with a minimum OD value of 0.3 reached by Fmoc-3,4F-Phe after 3 min, and by Fmoc-3,5F-Phe after 44 min (Figure 2c). Interestingly, the absorbance of Fmoc-3,4F-Phe subsequently increased gradually to reach an OD of 1, while the absorbance of the Fmoc-3,5F-Phe hydrogel remained stable for 16 h. Although the building blocks of both Fmoc-3,4F-Phe and Fmoc-3,5F-Phe can be self-assembled into nanofibers, the chemical bonds, arrangement and kinetics are significantly different. Apparently, in the Fmoc-3,4F-Phe the electron clouds create repulsion resulting in an unstable hydrogel which decays over time. In contrast, the lower electron rejection in the Fmoc-3,5F-Phe results in a more stable structure for a longer period of time.

### 2.2. Physical Characterization of Double-Fluorinated Fmoc-Phe Hydrogels

**Figure 3:**
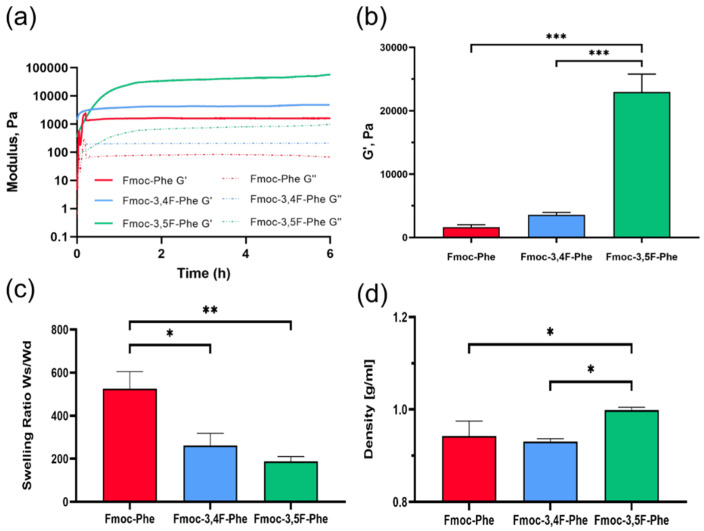
Physical Properties of Fmoc-Phe and the double fluorinated Fmoc-Phe hydrogels. (**a**) In situ time sweep oscillation measurements of storage and loss modulus. (**b**) The averaged storage modulus, G′, one-hour post-gelation. (**c**) Swelling ratio. (**d**) Density measurement. Representative results from three independent experiments are presented as mean ± SD; * *p* < 0.05, ** *p* < 0.01 and *** *p* < 0.001 as measured using one-way ANOVA.

Rheological analysis was performed at 25 °C to evaluate the mechanical properties of the hydrogels. First, dynamic strain sweep (5 Hz) and frequency sweep (0.5% strain) oscillatory measurements were performed to identify the appropriate conditions (Figure S2, Supporting Information). Based on the frequency sweep and oscillatory strain sweep analysis, the in-situ kinetics of the hydrogels formation and their mechanical properties were characterized by time sweep measurements at a fixed strain of 0.5% and frequency of 5 Hz, over 6 h (Figure 3). The storage modulus, G′, is typically significantly higher than the loss modulus, G″, of these hydrogels, which is indicative of a viscoelastic gel. In all cases, Tanδ (G″/G′) values after gelation were in the range of 0.05-0.015, i.e., <0.1, indicating stable hydrogel formation [35] (Figure S3, Supporting Information). 

The results of the rheological analysis revealed that the G′ of the Fmoc-Phe hydrogel, is relatively low, at 1600 Pa, whereas the Fmoc-3,4F-Phe has a slightly higher G′ value of 4800 Pa. In contrast, the Fmoc-3,5F-Phe hydrogel exhibits high rigidity, with a G′ value of more than 50,000 Pa. Significant differences in G′ values were observed by 1-hour post gelation (Figure 3b). At this time, the Fmoc-Phe hydrogel was entirely gelated with a G′ value of 1600 Pa, while the double fluorinated hydrogels had only achieved 40–70% of their end point storage modulus values of 3500 Pa and 23,000 Pa for Fmoc-3,4F-Phe and Fmoc-3,5F-Phe, respectively. The high storage modulus of the Fmoc-3,5F-Phe is probably due to stronger π–π interactions which contribute to the hydrogel stability, which caused by the higher distance between the fluorine atoms in comparison to Fmoc-3,4F-Phe. The relatively high mechanical rigidity of Fmoc-3,5F-Phe, combined with its high stability make the material interesting for tissue engineering and cell culture applications, where the mechanical properties are essential for controlling processes, such as stem cell differentiation. In this context, mesenchymal stem cells have been shown to undergo stiffness-directed fate differentiation into osteogenic lineages on rigid hydrogels [36]. 

The uptake of water into a hydrogel is very important because it can determine the overall permeation of nutrients and the excretion of cellular waste out of the hydrogel [37]. Whereas after 24 h in ddH_2_O, the Fmoc-3,5F-Phe hydrogel retained its initial 3D-shape with a swelling ratio of 187 Ws/Wd (Figure 3c), the Fmoc-3,4F-Phe disintegrated slightly and presented a higher swelling ratio of 262 Ws/Wd, and the Fmoc-Phe hydrogel broke apart completely with a very high swelling ratio of 525 Ws/Wd. Despite the importance of water absorption, high swelling can be a disadvantage and can make the material unsuitable for use as a scaffold in aqueous environments because it will not retain its 3D structure. The density of the double fluorinated Fmoc-Phe hydrogels was examined in order to provide information about the significant differences seen in water absorption. As expected, the Fmoc-3,5F-Phe hydrogel had a higher density than the others with a value of approximately 1 g mL^−1^ (Figure 3d). While Fmoc-3,4F-Phe and Fmoc-Phe have almost the same density of 0.93-0.94 g mL^−1^, the significant differences in swelling properties are probably due to the elasticity of the chains composing the hydrogel. It is possible that the chains in Fmoc-Phe are more elastic and flexible, which improves the water interaction and diffusion properties, while Fmoc-3,4F-Phe might form a more rigid fibrous matrix, which allows an exchange of water molecule at a steady state but limits the swelling [38]. Additionally, the lower electron rejection in the Fmoc-3,5F-Phe might allow for closer and denser interactions, contributing a denser packing which results in less swelling and higher mechanical properties.

The physical characterization highlights the advantages of these two double-fluorinated Fmoc-Phe hydrogels. Fmoc-Phe form a weak hydrogel which does not retain shape in an aqueous environment. Moreover, the Fmoc-F_5_-Phe form a weak hydrogel that is unstable over time, probably due to the large electron clouds derived from its five fluorine atoms. We have previously reported the improvement of the Fmoc-F_5_-Phe stability by forming a hybrid hydrogel with additional peptide [31]. Here we present how a single atom modification can form hydrogels which exhibit higher rigidity and stability over time, even in aqueous solution. It is shown that Fmoc-3,5F-Phe, in particular, remains very stable and shows improved physical properties compared to the other hydrogels.

### 2.3. Phase Transition and Morphological Characterization of the Assemblies

**Figure 4:**
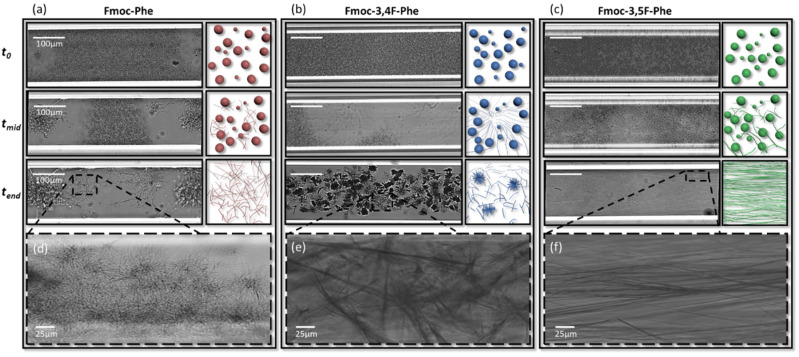
Real-time monitoring of the phase transition over time, and a schematic illustration of the different structures. Phase transition at three different time points; (*t*_0_) spherical structures, (*t_mid_*) the spheres disassembly slowly and are replaced by a fibrillary gel, (*t_end_*) final morphology of each building block (**a**) Fmoc-Phe (**b**) Fmoc-3,4F-Phe (**c**) Fmoc-3,5F-Phe. The final stage is shown at a magnified scale to enable visualization of the differences in morphology for (**d**) Fmoc-Phe (**e**) Fmoc-3,4F-Phe (**f**) Fmoc-3,5F-Phe.

Time-lapse optical microscopy measurements in real-time were used to monitor the kinetics and dynamics of the self-assembly process. The samples were sealed in a glass capillary to prevent evaporation, and based on our previous report of the Fmoc-F_5_-Phe phase transition [29], the experiment was performed in 50:50 DMSO:ddH_2_O solution. Interestingly, we also observed a phase transition for Fmoc-Phe and the double fluorinated Fmoc-Phe under these conditions (Figure 4, Movies S1–S3). Figure 4 presents real-time images and a schematic illustration of the phase transition of Fmoc-Phe and the two derivatives. At *t*_0_, all three building blocks exhibited a spherical structure, which over the next few minutes was gradually replaced by a fibrillary network (*t_mid_*). Interestingly, from that point on, the kinetics and morphologies of the various building blocks diverged, with Fmoc-Phe exhibiting multiple nucleation centers (Figure 4a,d and Movie S1). Initially, a similar process was observed for Fmoc-3,4F-Phe; however, a few minutes later, this continued to a third phase involving the growth of needle-like crystals (Figure 4b,e and Movie S2). In contrast to the other two building blocks, the assembled fibrils of Fmoc-3,5F-Phe are aligned along the capillary (Figure 4c,f and Movie S3). The disassembly and replacement of the spherical structures seen suggests that they are thermodynamically metastable compared to the gel and crystal phases. This is in accordance with previous reports concerning other supramolecular systems [29,39,40,41,42,43]. Our results demonstrate that the three different building blocks all exhibit a phase transition under the same conditions. These phase transitions are not reversible process, as the final phase is the preferred and stable thermodynamic state. This behavior appears to follow Ostwald’s rule of stages whereby monomers of metastable structural species can be replaced by a more stable phase such as fibrils and crystals. However, further analysis will be needed to obtain a deeper understanding of the kinetics as well as the thermodynamics of this process.

The results of the microscopy analysis are in accordance with the physical characterization of the structures. The low storage modulus obtained for the Fmoc-Phe hydrogel, as well as the low density and high swelling ratio, might be explained by the thick fibrillar morphology. In contrast, the thinner and more entangled fibrils of Fmoc-3,4F-Phe probably contribute to the higher storage modulus of this hydrogel. In addition, the curled fibrils could make it difficult for water to penetrate and thereby explain the lower swelling. Similarly, the thin, long, aligned nanofibrils seen in Fmoc-3,5F-Phe, might be directly responsible for the high storage modulus [44]. The straight orientation of the fibrils could be difficult to fold and reduce the flexibility, explaining how Fmoc-3,5F-Phe can be organized in a denser structure that exhibits less swelling. 

### 2.4. Structural Analysis by Powder and Single Crystal XRD

**Figure 5:**
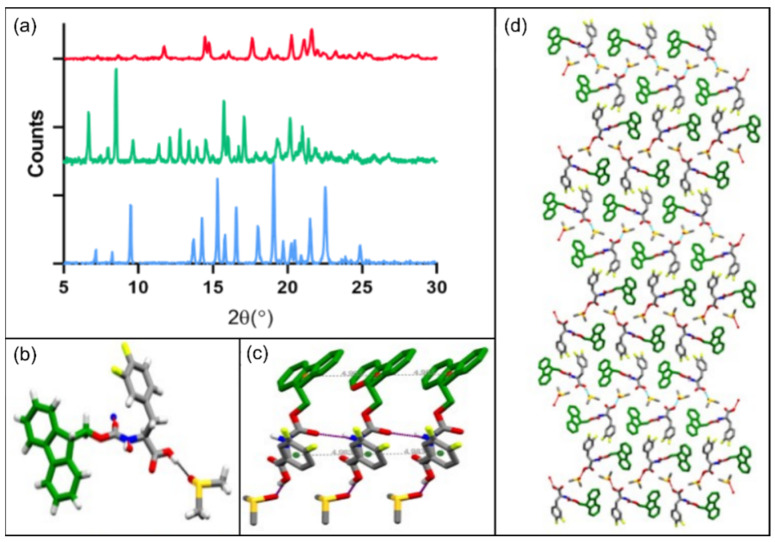
PXRD and Single crystal XRD structures. (**a**) PXRD of Fmoc-Phe (red) and the double fluorinated Fmoc-Phe building blocks; Fmoc-3,4F-Phe (blue), and Fmoc-3,5F-Phe (green). (**b**) Single crystal XRD structure of Fmoc-3,4F-Phe, demonstrating the asymmetric unit. (**c**) Intermolecular backbone hydrogen bonding and π–π stacking of the Fmoc-3,4F-Phe molecule, with a distance between two centroids of 4.983 Å. (**d**) Fmoc-3,4F-Phe crystal packing along the c-axis.

Powder XRD (PXRD) was used to study the molecular organization of the different building blocks further. The PXRD pattern of Fmoc-Phe, Fmoc-3,4F-Phe and Fmoc-3,5F-Phe presented in Figure 5a includes peaks for all the samples, indicating their crystalline nature. However, Fmoc-3,4F-Phe and Fmoc-3,5F-Phe exhibit sharper and higher intensity peaks, which indicate a higher degree of crystallization. The diffraction patterns of Fmoc-3,4F-Phe and Fmoc-3,5F-Phe are different, which implies two distinct crystalline arrangements. 

Using single crystal XRD, we were able to solve the crystal structure of Fmoc-3,4F-Phe for the first time, and detailed crystallographic data are presented in Table S1 in the Supporting Information. This analysis revealed that the Fmoc-3,4F-Phe crystallizes in the orthorhombic space group P2_1_2_1_2_1_ with one Fmoc-3,4F-Phe and one co-crystallized DMSO solvent molecule in the asymmetric unit (Figure 5b). Careful observation of the crystal structure indicated that the backbone torsional angle φ and ψ have a value near −130° and 165°, which fall in the extended sheet region of the Ramachandran plot. More importantly, the carbamate group of Fmoc-3,4F-Phe is connected to the neighboring molecule through an intermolecular hydrogen bond (N–H···O). In addition, the carboxylate group of each Fmoc-3,4F-Phe forms an intermolecular hydrogen bond (O–H···O) with the DMSO solvent molecule. The face-to-face π–π interactions between the Fmoc-Fmoc groups of the subunit, together with π–π interactions between the side chain 3,4F-Phe groups and a neighboring molecule, stabilize the overall packing (Figure 5c). 

Interestingly, weak intermolecular C–H···F hydrogen bonds were observed between the subunits, and the molecule further self-organized to produce a helical like architecture along the crystallographic c-axis in higher-order packing (Figure 5d). This helical like architecture correlates with the presence of chiral carbons in the Fmoc-3,4F-Phe structure, and represents a generic structural motif in self-assembled amino acid and peptide nanostructures [45]. In contrast to the orthorhombic space group P2_1_2_1_2_1_ crystals seen for Fmoc-3,4F-Phe, Fmoc-Phe crystallized in the monoclinic space group P2_1_ [26,27,46,47]. The Fmoc-Phe crystal structure exhibits strong intermolecular hydrogen bonding N1–H1···O2 between Fmoc-Phe molecules, and strong π–π stacking interactions between the large bulky Fmoc group, as well as between phenylalanines, in a similar scenario to that observed in the Fmoc-3,4F-Phe crystal structure. However, while the carboxylic acid group of Fmoc-Phe forms intermolecular hydrogen bonds with the adjacent carboxylic group of another Fmoc-Phe molecule, the carboxylic acid group of Fmoc-3,4F-Phe undergoes intermolecular hydrogen bonding with a DMSO solvent molecule. Importantly, this difference in intermolecular hydrogen bonding, means that Fmoc-3,4F-Phe has significantly higher order packing than Fmoc-Phe.

### 2.5. Structural Analysis by MD Simulations 

After solving the structure of Fmoc-3,4F-Phe by single crystal XRD analysis, and without success in crystalizing Fmoc-3,5F-Phe, we further explored the structural differences between the two molecules through MD simulations. The MD simulations were used to independently study and compare the properties of Fmoc-3,4F-Phe and Fmoc-3,5F-Phe during the first moments of self-assembly without any bias from XRD results. Five-replicate explicit solvent (water) MD simulations, followed by structural analyses of the resulting MD simulation trajectories, for each derivative were performed. Both derivatives exhibited gradual formation of aggregates, which may represent the initial organization of Fmoc-3,4F-Phe and Fmoc-3,5F-Phe. We note that the interactions and statistical trends described below are reproducible across all MD simulations despite the formation of different aggregate structures within MD simulation replicates.

**Figure 6:**
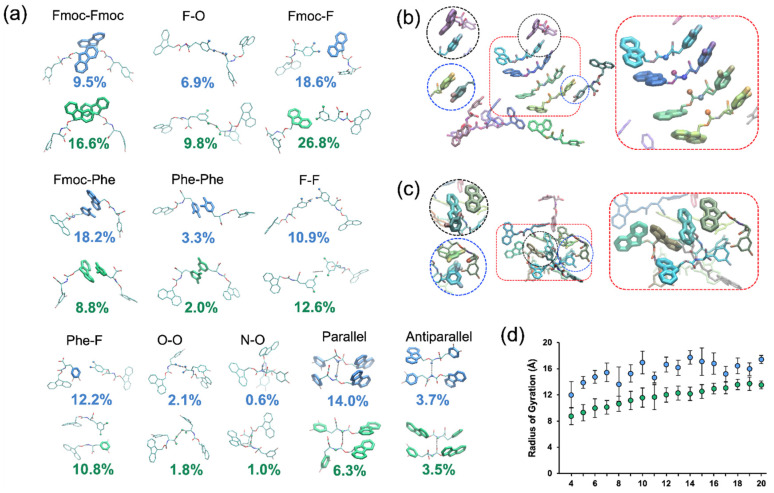
MD simulations depicting the structural properties of the Fmoc-3,4F-Phe and Fmoc-3,5F-Phe assemblies. (**a**) Molecular graphics images of interactions between (top row, blue) Fmoc-3,4F-Phe and (bottom row, green) Fmoc-3,5F-Phe pairs observed in simulations and their average percent frequency across all MD simulations. Percent frequency was calculated as the total number of instances for which a given interaction was observed, divided by the total number of interactions observed in each simulation. The average standard deviation of the average percent frequencies is 0.9 ± 0.6% with a minimum and maximum standard deviation of 0.1 and 2.6%, respectively; the relatively small values indicate good reproducibility across different simulation runs per system. (**b**,**c**) Representative aggregate of 10 (**b**) Fmoc-3,4F-Phe and (**c**) Fmoc-3,5F-Phe building-block monomers observed in the simulations with key differences in interactions encircled in dotted lines and zoomed-in in the nearby panels. Molecular graphics images in (**a**–**c**) were produced using Visual Molecular Dynamics (VMD) version 1.9.4 [50]. (**d**) Radius of gyration (Å) of building block-monomers within the clusters observed in the simulations of Fmoc-3,4F-Phe (blue) and Fmoc-3,5F-Phe (green).

The results of the MD simulations indicate that the aggregates formed by self-assembly of Fmoc-3,4F-Phe or Fmoc-3,5F-Phe are significantly stabilized by π–π interactions between aromatic groups as well as F-F and F-Phe contacts (Figure 6a–c). In addition, both derivatives interacted occasionally through hydrogen bonds between terminal carboxyl groups or backbone amide and carbonyl groups of opposing monomers. 

Our analysis revealed similarities and differences in the structural properties of the two derivatives as a result of the interactions formed within the simulated clusters (Figure 6a). While both Fmoc-3,4F-Phe and Fmoc-3,5F-Phe formed structures reminiscent of an antiparallel β-sheet at a similar rate (Figure 6a), Fmoc-3,4F-Phe formed a parallel β-sheet-like structure, in which two monomers are bonded through a hydrogen bond between their backbone amide and carbonyl groups as well as face-to-face π–π interactions between the Fmoc-Fmoc or Phe-Phe groups (Figure 6b, boxed in red dotted lines) more often than Fmoc-3,5F-Phe. Importantly, this ordered structure is reminiscent of the crystal structure of Fmoc-3,4F-Phe (Figure 5c and Figure 6b boxed in red dotted lines). The preferential ability of Fmoc-3,4F-Phe to form face-to-face π–π interactions between Fmoc-Phe and Phe-Phe groups (Figure 6b, boxed in black and blue dotted lines, respectively) could facilitate the formation of the parallel β-sheet-like structures observed in the MD simulations and crystal structure (Figure 5c and Figure 6b boxed in red dotted lines). In contrast, Fmoc-3,5F-Phe aggregates were more frequently stabilized by Fmoc-Fmoc π-stacking interactions, and contacts between fluorine of the 3,5F-Phe group and Fmoc, in the absence of Fmoc-Phe π-stacking, as well as interactions between the fluorine of the 3,5F-Phe group and the terminal O of opposing monomers (Figure 6c boxed in red, black, and blue dotted lines respectively). 

In addition to the differences in the frequency of interactions formed within their aggregates, we also observed that the calculated radius of gyration of the monomers within the clusters formed by Fmoc-3,5F-Phe was consistently lower than for those formed by Fmoc-3,4F-Phe. This was true across aggregates of different sizes (Figure 6d). Thus, for clusters containing the same number of building block-monomers, clusters formed by Fmoc-3,5F-Phe were more densely packed. This supports the experimental density measurements and provides additional evidence for differences in the self-assembly properties of the Fmoc-3,4F-Phe and Fmoc-3,5F-Phe systems.

Solvent exposure calculations of the Fmoc moiety and Phe sidechain of Fmoc-3,4F-Phe and Fmoc-3,5F-Phe within the independent detected clusters indicated that the Fmoc moiety is generally less solvent exposed in the clusters formed by Fmoc-3,5F-Phe compared to those formed by Fmoc-3,4F-Phe (Figure S4, orange and blue data points, respectively). In addition, the Phe sidechain is generally more solvent exposed in the clusters formed by Fmoc-3,5F-Phe compared to those formed by Fmoc-3,4F-Phe (Figure S4, yellow and grey data points, respectively). Thus, the Fmoc moiety is buried more deeply within the clusters of Fmoc-3,5F-Phe than in the clusters of Fmoc-3,4F-Phe, and the Phe sidechain is less buried within the clusters of Fmoc-3,5F-Phe than in the clusters of Fmoc-3,4F-Phe. This suggests that the different position of the fluorine atom in Fmoc-3,4F-Phe compared to Fmoc-3,5F-Phe may influence the hydrophobicity of the Phe sidechain. Specifically, as 3,4F-Phe is less solvent exposed than 3,5F-Phe within the detected clusters, 3,4F-Phe appears to be more hydrophobic than 3,5F-Phe. This is supported by polar desolvation energy calculations performed in AMSOL [48] for the isolated 3,4F-Phe and 3,5F-Phe sidechains, predicting that 3,5F-Phe should be less hydrophobic than 3,4F-Phe [49]. This difference in hydrophobicity between 3,4F-Phe and 3,5F-Phe provides an additional potential explanation for the differences in the interactions formed within the clusters of Fmoc-3,4F-Phe and the clusters of Fmoc-3,5F-Phe (Figure 6a,b).

## 3. Materials and Methods

### 3.1. Materials

Fmoc-L-Phe was purchased from Sigma-Aldrich (Israel), Fmoc-3,4F-Phe and Fmoc-3,5F-Phe were purchased from Chem-Implex Int’l inc (IL, USA).

### 3.2. Preparation of Fmoc-Phe Derivatives Self-Assemblies

Three different techniques were used for the preparation of Fmoc-Phe derivatives self-assemblies: Thermal-Switch, pH-Switch and Solvent-Switch. ***Thermal Switch***: Preformed structures were assembled by dissolving Fmoc-Phe derivatives in ddH_2_O at concentration of 5 g L^−1^ and heating to 90 °C. Structures were visible when samples were slowly cooled down to room temperature. ***pH Switch***: Fmoc-Phe derivatives were mixed in ddH_2_O at concentration of 5 g L^−1^ and sonicated until dissolved. A solution of 0.5 M NaOH was slowly added to the peptide until pH 7.5 was measured. The solution was left undisturbed until gelation was observed. ***Solvent Switch*** [51]: Stock solution were prepared in dimethyl sulfoxide (DMSO) or absolute ethanol (EtOH) at a concentration of 100 g L^−1^ and 10 g L^−1^, respectively. The hydrogels were formed by diluting the stock solution with ddH_2_O, resulting in a final concentration of 5 g L^−1^. 

### 3.3. OD Kinetics Analysis

Immediately after hydrogels preparation, 100 μL samples were placed into a 96-well plate. Absorbance and kinetics were measured at a wavelength of 350 nm using a TECAN Infinite M200PRO plate reader for 16 h.

### 3.4. Optical Microscopy Analysis

Samples of the three building blocks were prepared at a ratio of 50:50 DMSO:ddH_2_O and transferred into a thin glass capillary (0.2 mm inner diameter, 0.1 mm wall) and sealed to avoid evaporation. The capillary was attached to a glass slide and observed under an optical microscope. Bright-field imaging was performed using an Eclipse Ti-E inverted microscope (Nikon, Japan), equipped with a Zyla scMOS camera (Andor, UK). For the phase transition kinetics experiment, time-lapse image series were acquired using a 20× objective over time, with 10 s interval.

### 3.5. TEM

Hydrogels samples were placed on a 400-mesh copper grid. After 1 min, the piece of gel as well as the excess fluid was removed. Negative staining was obtained by covering the grid with 10 μL of 2% uranyl acetate in water. After 2 min, excess uranyl acetate solution was removed. Samples were viewed using a JEOL 1200EX electron microscope operating at 80 kV.

### 3.6. Rheology Analysis

In situ hydrogel formation, mechanical properties, and kinetics were characterized using an AR-G2 rheometer (TA Instruments). Time-sweep oscillatory tests in 20 mm parallel plate geometry were performed on 220 μL fresh solution (resulting in a gap size of 0.6 mm) at room temperature. Oscillatory strain (0.01–100%) and frequency sweeps (0.1–100 Hz) were conducted to find the linear viscoelastic region, in which the time-sweep oscillatory tests were performed (Figure S2, Supporting information). G′ and G″, the storage and loss moduli, respectively, were obtained at 5 Hz oscillation and 0.5% strain deformation for each sample. 

### 3.7. Swelling

Identical volumes of hydrogel samples were placed on plates. Five samples were used for each hydrogel. The initial weight (Wi) was recorded, and the hydrogels were placed in ddH_2_O. To allow equilibration and swelling, all samples were left to swell for 24 h on an orbital shaker (50 rpm) at room temperature. The equilibrated swollen mass (Ws) was recorded after gently absorbing excess water from each sample. The hydrogel samples were subsequently lyophilized, and their dry weight (Wd) was measured. The equilibrated swelling ratio (Q) was defined as the ratio of Ws to Wd. 

### 3.8. Density

The density measurement was conducted using a pycnometer at 23 °C. First, we filled the pycnometer with ddH_2_O and calculated the exact volume of each 5 ml pycnometer (Vp). Then we placed 500 µL hydrogel sample inside the pycnometer, weighed it (Ms) and waited an hour until complete gelation. We gently added ddH_2_O and calculate the ddH_2_O volume inside the pycnometer (Vd). Sample density was calculating by dividing Ms to (Vp-Vd).

### 3.9. Crystallization, Data Collection and Structure Determination

Fmoc-3,4F-Phe 100 g L^−1^ stock solutions were prepared in DMSO and diluted into ddH_2_O at a 70:30 ratio. The samples were left, half sealed, on the bench at room temperature, and crystals grew within 4 months. For data collection, a crystal was coated in Paratone oil (Hampton Research), mounted on a MiTeGencryo-loop, and flash-frozen in liquid nitrogen. Crystal data were measured at 100 K on a RigakuXtaLab^Pro^ diffractometer equipped with CuKα radiation (λ = 1.54184 Å) and a Dectris Pilatus3R 200K-A detector. The data were processed with CrysAlisPro programs (RigakuOD). The structure was solved by direct methods with SHELXT-2016/4 and refined with full-matrix least squares refinement based on F2 with SHELXL-2016/4. The crystallographic data have been deposited in the Cambridge Crystallographic Data Centre (CCDC) under no. 2043733 and are presented in supplementary information Table S1, Supporting Information.

### 3.10. Molecular Modeling of Investigated Systems

Fmoc-3,4F-Phe and Fmoc-3,5F-Phe were computationally modeled independently for use as initial structures in MD simulations, described in the next section, analogously to refs [52,53]. For each system, 36 monomers (either Fmoc-3,4F-Phe or Fmoc-3,5F-Phe) were embedded in a 40 × 40 × 60 Å^3^ grid within a 90 × 90 × 90 Å^3^ water box, with an equal spacing of approximately 20 Å between each monomer and random configurations and orientations. The random configurations were extracted from short simulations of each monomer at infinite dilution. The derivative concentration within each system was higher than the concentration used in the experimental studies, aiming to artificially accelerate the self-assembly within the frame of the simulations [52,53]. 

### 3.11. MD Simulations

The self-assembly of Fmoc-3,4F-Phe and Fmoc-3,5F-Phe were independently investigated through MD simulations analogously to refs [52,53]. Prior to the execution of MD simulations, the simulation systems were first energetically minimized and equilibrated. In the energetic minimization stage, 50 steps of steepest descent followed by 50 steps of Adopted Basis Newton-Raphson energy minimization were first performed with the monomers initially fixed to their initial conformations to alleviate clashes primarily between the monomers and their surrounding environment. Subsequently, an additional 100 steps of steepest descent followed by 100 steps of Adopted Basis Newton-Raphson energy minimization were performed with the monomer heavy atoms were constrained with 0.1 kcal mol^−1^ Å^−2^. Under the same constraints, after the energetic minimization, the system was equilibrated for 1 ns. Finally, five replicate MD simulation production runs with different initial velocities were performed for each system. Multiple serial simulation runs were preferred over other single simulation runs using enhanced sampling methods, as routinely used by Tamamis and co-authors [52,54,55,56,57,58], at the same computational cost as the former were used advantageously to check reproducibility of the results in the current study. Here, reproducibility was considered important to delineate the differences in self-assembly between the two systems with subtle structural differences at the monomeric state. In the MD simulation production runs, no constraints were imposed on the system. All energy minimization and MD simulations were performed using periodic boundary conditions and the CHARMM36 force field [59] in CHARMM [60]. Parameters and topologies for the derivatives were generated using CGenFF [61]. The temperature and pressure of the simulation systems was maintained at 300 K and 1.0 atm using the dual Nosé-Hoover thermostat and the Andersen-Hoover barostat, respectively. The bond lengths of covalently bonded hydrogens were constrained using the SHAKE algorithm [62]. 

### 3.12. Structural Analysis of MD Simulations

Both Fmoc-3,4F-Phe and Fmoc-3,5F-Phe were observed to form aggregates within their respective sets of MD simulations. We aimed to investigate the early-stage gradual formation of structural morphologies in the two systems, and thus, we simulated and analyzed 80 ns of each replicate in both systems under investigation, which corresponds to the time at which the systems started forming rather stable aggregates based on radius of gyration calculations. Simulation snapshots were extracted in 0.2 ns intervals per simulation production run, resulting in 2000 analyzed snapshots per system. Structural analysis programs were to understand and compare the structural organization properties of the two derivatives, independently, in the first moments of self-assembly. Crystallographic data of Fmoc-3,4F-Phe and visual inspection of the MD simulations were used to guide the definitions of key interactions that are formed in ordered assemblies of Fmoc-3,4F-Phe and Fmoc-3,5F-Phe, independently, in their respective simulations. The interactions detected are shown in Figure 6a. A uniform distance cutoff value of 4.0 Å was used as a criterion to define an interaction between the atoms described in Figure 6a. The same set of interactions was tracked for both the simulations of Fmoc-3,4F-Phe and Fmoc-3,5F-Phe for a fair comparison of the two derivatives’ self-assembly properties. A number of *n* monomers were defined to form a cluster when each building block-monomer was in the vicinity of at least another one based on the 4.0 Å distance criterion defined above.

We additionally calculated the radius of gyration of the building block-monomers within each observed cluster to determine and compare the compactness of the clusters formed by Fmoc-3,4F-Phe to those formed by Fmoc-3,5F-Phe (Figure 6d). The radius of gyration of the building block-monomers within each cluster was calculated using the following equation in Wordom: [63,64]

$$R_{g}=\sqrt{\frac{1}{N}}\sum_{k=1}^{N}{\left(r_{k}-\bar{r}\right)}^{2}$$

In the aforementioned equation, the radius of gyration, *R_g_*, is calculated as the square root of the average deviation of *N* atoms, *r_k_*, from the geometric center, *r*. The radius of gyration calculations was performed considering only building block-monomers within each cluster, with all other atoms omitted. Larger relative radius of gyration values indicates lower compactness, or lower packing density of building blocks in the clusters, while lower relative radius of gyration values indicates higher compactness, or higher density of building blocks in the clusters [55]. 

Finally, we calculated the solvent exposure of the Fmoc-moiety and modified Phe side chains within each observed cluster to determine and compare on how the aromatic rings of Fmoc-3,4F-Phe and Fmoc-3,5F-Phe were positioned (in terms of “burial” or not) within each of their respective clusters (Figure S4). The solvent exposition of the Fmoc-moiety and modified Phe side chains, independently, of the building block-monomers within each cluster was calculated as the solvent accessible surface area (SASA) of the Fmoc-moiety or modified Phe side chain divided by the total molecular surface area (TSA) of the same Fmoc-moiety or modified Phe side chain. The SASA and TSA values for each Fmoc-moiety or modified Phe side chain was calculated in Wordom [63,64]. A larger percent solvent exposure of a Fmoc-moiety or modified Phe side chain indicates that the moiety or side chain is more exposed to the solvent and more likely to be at the surface of the cluster; a smaller the percent solvent exposure of a Fmoc-moiety or modified Phe side chain indicates that the moiety or side chain is more “buried” and more likely to be encapsulated in the interior of the cluster.

### 3.13. Statistical Analysis

Statistical significance was examined using one-way ANOVA to determine the *p*-value. *p* < 0.05 was considered a statistically significant difference

## 4. Conclusions

The current study provides the first description of the behavior of the double-fluorinated Fmoc-Phe derivatives, Fmoc-3,4F-Phe and Fmoc-3,5F-Phe, and the effect that the position of a single fluorine has on the self-assembly process, and physical properties that the material produces. Our results reveal substantial differences between the derivatives. While Fmoc-3,5F-Phe is transparent and remains clear and stable over time, both Fmoc-Phe and Fmoc-3,4F-Phe disintegrate and undergo phase separation. Moreover, Fmoc-3,5F-Phe presents a more orderly and aligned microstructure of nanofibrils and poses a higher storage modulus, and lower swelling due to a higher density. Although all three building blocks exhibit a spontaneous phase transition process from metastable spheres to fibrils, Fmoc-3,4F-Phe undergoes an additional self-assembly event, resulting in the formation of crystals. Single crystal XRD of the Fmoc-3,4F-Phe crystal structure revealed that π–π interactions and hydrogen bonding contribute to the crystal stability. MD simulations provided additional evidence of differences between the structural properties of Fmoc-3,4F-Phe and Fmoc-3,5F-Phe. While Fmoc-3,4F-Phe aggregates are more frequently stabilized through interactions reminiscent of the crystal structure, the stability of Fmoc-3,5F-Phe aggregates rely more on Fmoc-Fmoc π stacking, contacts between the F of the 3,5F-Phe group and Fmoc, as well as interactions between the F of the 3,5F-Phe group and the terminal O of the opposing monomer. The experimental data and the simulations both indicate that Fmoc-3,5F-Phe forms more compact aggregates than Fmoc-3,4F-Phe. These results highlight the effect of the position of a single amino acid on the self-assembly process and demonstrate that fluorination is an effective strategy to influence nanoscale supramolecular organization. Our results are consistent with the changes in the Phe fluorination pattern observed in previously published work and emphasize the often-unpredicted consequences of minor change in the building block structure that complicate rational design of such materials. They also demonstrate how the macroscale properties of a material can be modified by atomic scale changes in the constituent molecules. Furthermore, they provide fundamental insights that will facilitate the development of optimal amino-acid-based low-molecular-weight hydrogelators for a wide range of applications in various fields. 

## Data Availability

CCDC 2043733 contains the supplementary crystallographic data for this paper. These data can be obtained free of charge from the Cambridge Crystallographic Data Centre via www.ccdc.cam.ac.uk/data_request/cif (accessed on 30 August 2021).

## Supplementary Materials

The following are available online at https://www.mdpi.com/article/10.3390/ijms22179634/s1.
