# Supplementary material for: Modification of a Single Atom Affects the Physical Properties of Double Fluorinated Fmoc-Phe Derivatives
Source: Int J Mol Sci. 2021 Sep 6;22(17):9634. doi: 10.3390/ijms22179634 (PMC8431810; doi:10.3390/ijms22179634)

checkCIF/PLATON report

Structure factors have been supplied for datablock(s) lihi2f

THIS REPORT IS FOR GUIDANCE ONLY. IF USED AS PART OF A REVIEW PROCEDURE FOR PUBLICATION, IT SHOULD NOT REPLACE THE EXPERTISE OF AN EXPERIENCED CRYSTALLOGRAPHIC REFEREE.

No syntax errors found. CIF dictionary Interpreting this report

Datablock: lihi2f

```
Bond precision:  C-C = 0.0053 A          Wavelength=1.54184

Cell:             a=4.9826(1)     b=13.0717(2)     c=36.5189(5)
                  alpha=90        beta=90          gamma=90
Temperature:      100 K

                Calculated                 Reported
Volume          2378.52(7)                 2378.51(7)
Space group     P 21 21 21                 P 21 21 21
Hall group      P 2ac 2ab                  P 2ac 2ab
Moiety formula  C24 H19 F2 N O4, C2 H6 O S C24 H19 F2 N O4, C2 H6 O S
Sum formula     C26 H25 F2 N O5 S          C26 H25 F2 N O5 S
Mr              501.53                     501.53
Dx,g cm-3       1.401                      1.401
Z               4                          4
Mu (mm-1)       1.684                      1.684
F000            1048.0                     1048.0
F000'           1052.79
h,k,lmax        6,16,46                    6,16,45
Nref            5191[ 3044]                4854
Tmin,Tmax       0.980,0.983                0.556,1.000
Tmin'           0.714

Correction method= # Reported T Limits: Tmin=0.556 Tmax=1.000
AbsCorr = MULTI-SCAN

Data completeness= 1.59/0.94      Theta(max)= 79.703

R(reflections)= 0.0550( 4634)     wR2(reflections)= 0.1533( 4854)

S = 1.057                  Npar= 342
```

The following ALERTS were generated. Each ALERT has the format
test-name_ALERT_alert-type_alert-level.
Click on the hyperlinks for more details of the test.

Alert level C

```
DIFMX02_ALERT_1_C  The maximum difference density is > 0.1*ZMAX*0.75
            The relevant atom site should be identified.
PLAT094_ALERT_2_C Ratio of Maximum / Minimum Residual Density ....       3.72 Report
PLAT097_ALERT_2_C Large Reported Max.  (Positive) Residual Density       1.42 eA-3
PLAT213_ALERT_2_C Atom O3              has ADP max/min Ratio .....        3.1 prolat
PLAT213_ALERT_2_C Atom O4              has ADP max/min Ratio .....        3.2 prolat
PLAT220_ALERT_2_C NonSolvent Resd 1  O   Ueq(max) / Ueq(min) Range        4.3 Ratio
PLAT222_ALERT_3_C NonSolvent Resd 1  H   Uiso(max)/Uiso(min) Range        5.1 Ratio
PLAT242_ALERT_2_C Low    'MainMol' Ueq as Compared to Neighbors of        C17 Check
PLAT340_ALERT_3_C Low Bond Precision on  C-C Bonds ..............     0.00532 Ang.
PLAT911_ALERT_3_C Missing FCF Refl Between Thmin & STh/L=    0.600         10 Report
PLAT915_ALERT_3_C No Flack x Check Done: Low Friedel Pair Coverage         87 %
```

Alert level G

```
PLAT002_ALERT_2_G Number of Distance or Angle Restraints on AtSite          5 Note
PLAT003_ALERT_2_G Number of Uiso or Uij Restrained non-H Atoms ...          2 Report
PLAT007_ALERT_5_G Number of Unrefined Donor-H Atoms .............           2 Report
PLAT173_ALERT_4_G The CIF-Embedded .res File Contains DANG Records          1 Report
PLAT176_ALERT_4_G The CIF-Embedded .res File Contains SADI Records          1 Report
PLAT186_ALERT_4_G The CIF-Embedded .res File Contains ISOR Records          1 Report
PLAT302_ALERT_4_G Anion/Solvent/Minor-Residue Disorder (Resd  2  )        50% Note
PLAT413_ALERT_2_G Short Inter XH3 .. XHn     H11      ..H29B     .       2.14 Ang.
                                        -x,-1/2+y,3/2-z  =      3_546 Check
PLAT791_ALERT_4_G Model has Chirality at C16         (Sohnke SpGr)          S Verify
PLAT860_ALERT_3_G Number of Least-Squares Restraints ............          20 Note
PLAT883_ALERT_1_G No Info/Value for _atom_sites_solution_primary .     Please Do !
PLAT910_ALERT_3_G Missing # of FCF Reflection(s) Below Theta(Min).          1 Note
PLAT912_ALERT_4_G Missing # of FCF Reflections Above STh/L=  0.600         54 Note
PLAT933_ALERT_2_G Number of OMIT Records in Embedded .res File ...          6 Note
PLAT941_ALERT_3_G Average HKL Measurement Multiplicity ..........         3.8 Low
PLAT978_ALERT_2_G Number C-C Bonds with Positive Residual Density.          2 Info
```

0 **ALERT level A** = Most likely a serious problem - resolve or explain
0 **ALERT level B** = A potentially serious problem, consider carefully
11 **ALERT level C** = Check. Ensure it is not caused by an omission or oversight
16 **ALERT level G** = General information/check it is not something unexpected

2 ALERT type 1 CIF construction/syntax error, inconsistent or missing data
11 ALERT type 2 Indicator that the structure model may be wrong or deficient
7 ALERT type 3 Indicator that the structure quality may be low
6 ALERT type 4 Improvement, methodology, query or suggestion
1 ALERT type 5 Informative message, check

It is advisable to attempt to resolve as many as possible of the alerts in all categories. Often the minor alerts point to easily fixed oversights, errors and omissions in your CIF or refinement strategy, so attention to these fine details can be worthwhile. In order to resolve some of the more serious problems it may be necessary to carry out additional measurements or structure refinements. However, the purpose of your study may justify the reported deviations and the more serious of these should normally be commented upon in the discussion or experimental section of a paper or in the "special_details" fields of the CIF. checkCIF was carefully designed to identify outliers and unusual parameters, but every test has its limitations and alerts that are not important in a particular case may appear. Conversely, the absence of alerts does not guarantee there are no aspects of the results needing attention. It is up to the individual to critically assess their own results and, if necessary, seek expert advice.

Publication of your CIF in IUCr journals

A basic structural check has been run on your CIF. These basic checks will be run on all CIFs submitted for publication in IUCr journals (*Acta Crystallographica*, *Journal of Applied Crystallography*, *Journal of Synchrotron Radiation*); however, if you intend to submit to *Acta Crystallographica Section C* or *E* or *IUCrData*, you should make sure that full publication checks are run on the final version of your CIF prior to submission.

Publication of your CIF in other journals

Please refer to the *Notes for Authors* of the relevant journal for any special instructions relating to CIF submission.

PLATON version of 04/06/2020; check.def file version of 02/06/2020

Datablock lihi2f - ellipsoid plot

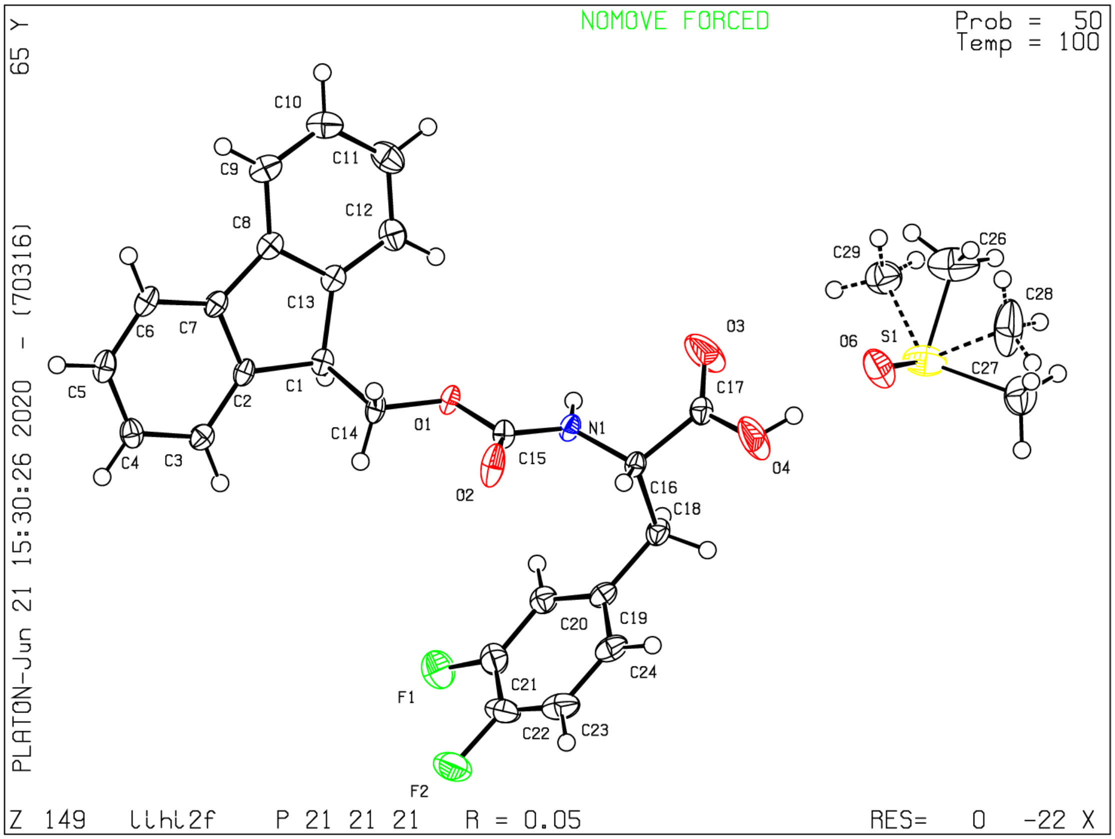

Supplement: Supplementary file 1 [file ijms-22-09634-s001.zip › FmocF3,4-checkcif.pdf]
