# Supplementary material for: Modification of a Single Atom Affects the Physical Properties of Double Fluorinated Fmoc-Phe Derivatives
Source: Int J Mol Sci. 2021 Sep 6;22(17):9634. doi: 10.3390/ijms22179634 (PMC8431810; doi:10.3390/ijms22179634)
Supplement: Supplementary file 1 [file ijms-22-09634-s001.zip › ijms-1331669-supple after proof.pdf]

Supporting Information

Modification of a Single Atom Affects the Physical Properties of Double Fluorinated Fmoc-Phe Derivatives

Moran Aviv[a,b+], Dana Cohen-Gerassi[a,c+], Asuka A. Orr[d], Rajkumar Misra[a], Zohar A. Arnon[a], Linda J W Shimon[e], Yosi Shacham-Diamand[c,f,g], Phanourios Tamamis[d,h], and Lihi Adler-Abramovich[a]*

[a]Department of Oral Biology, The Goldschleger School of Dental Medicine, Sackler Faculty of Medicine, The Center for Nanoscience and Nanotechnology, The Center for the Physics and Chemistry of Living Systems, Tel Aviv University, Tel Aviv 6997801, Israel

[b]School of Mechanical Engineering, Afeka Tel Aviv Academic College of Engineering, Tel Aviv 6910717, Israel

[c]Department of Materials Science and Engineering, Iby and Aladar Fleischman Faculty of Engineering, Tel Aviv University, Tel Aviv 6997801, Israel

[d]Department of Chemical Engineering, Texas A&M University, College Station, Texas 77843-3122, United States.

[e]Department of Chemical Research Support, Weizmann Institute of Science, Rehovot, 76132701, Israel

[f]Department of Physical Electronics, School of Electrical Engineering, Faculty of Engineering, Tel Aviv University, Tel-Aviv 69978, Israel.

[g]Endowed Chair Professor, TAU/TiET Food Security Center of Excellence (T2FSCoE), Direcrtor, Thapar In-stitute of Engineering and Technology, Patiala, Punjab, India.

[h]Department of Materials Science and Engineering, Texas A&M University, College Station, Texas 77843-3122, United States.

[*]Corresponding author: lihia@tauex.tau.ac.il

[+]M.A. and D.C.-G. contributed equally to this work.

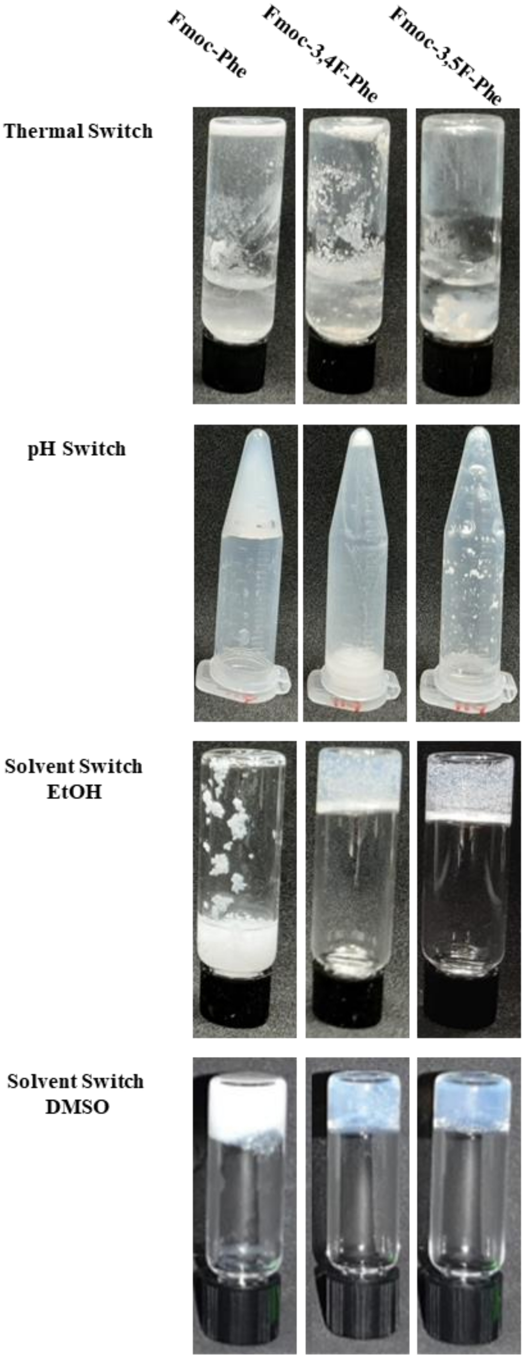

Figure S1 Fmoc-Phe derivatives (5 g L^{-1}) one-hour post preparation at different self-assembly methods.

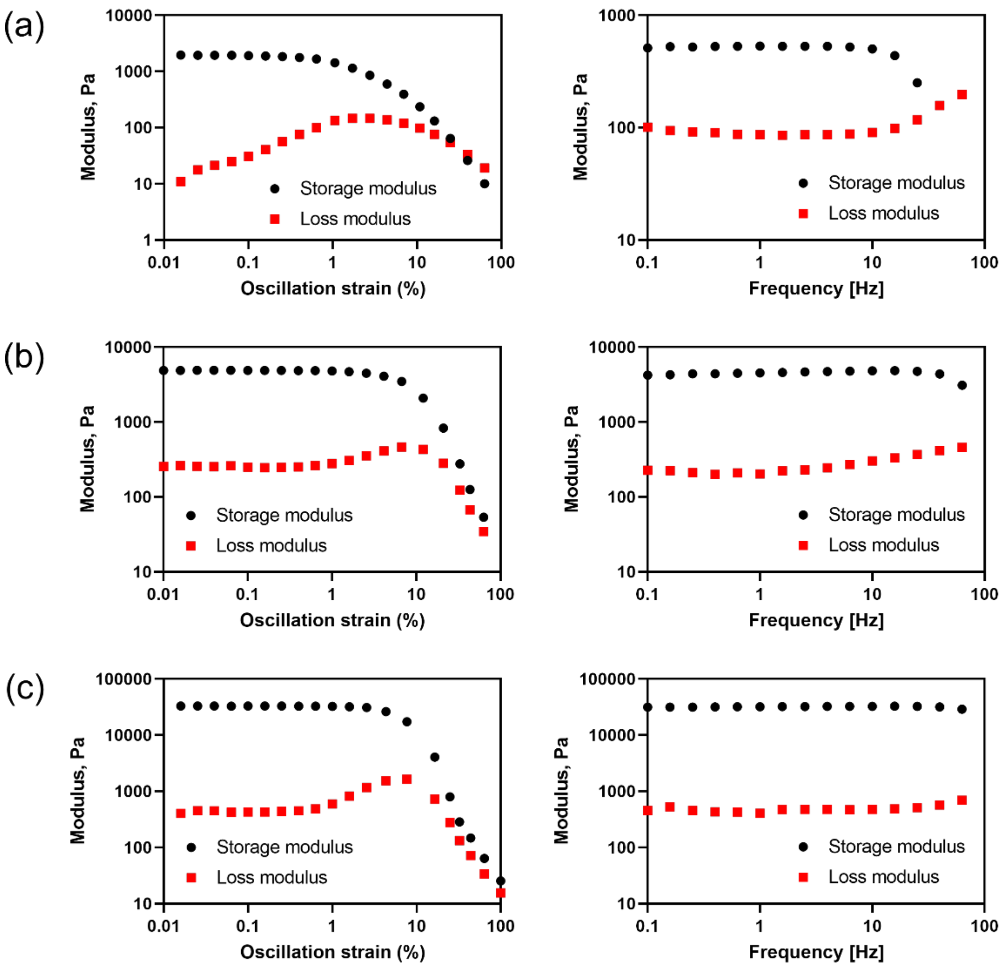

Figure S2 Rheological characterization of strain sweep at 5 Hz (Left) and frequency sweep at 0.5% strain (Right) of (a) Fmoc-Phe (b) Fmoc-3,4F-Phe (c) Fmoc-3,5F-Phe.

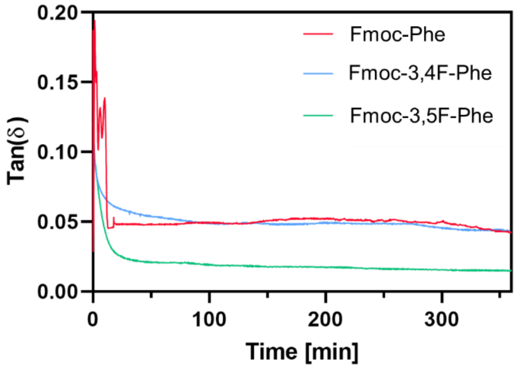

Figure S3 Tan (δ) values of Fmoc-Phe and double fluorinated Fmoc-Phe hydrogels.

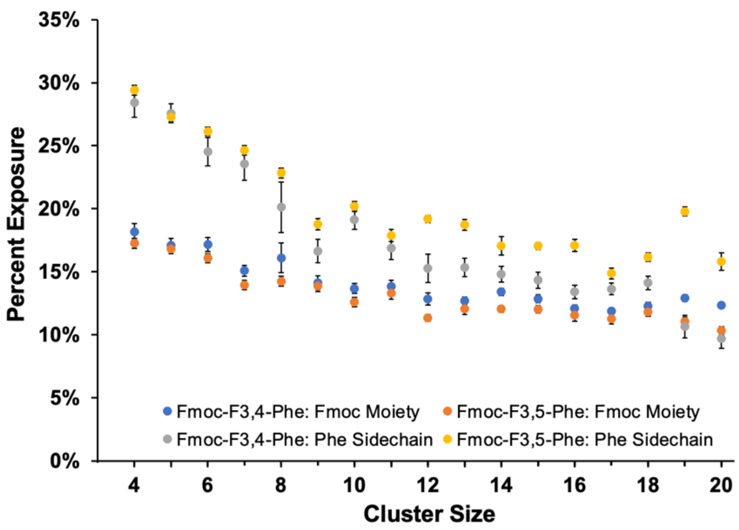

Figure S4 Percent solvent exposures of the Fmoc moiety (blue and orange) and Phe sidechain (grey and yellow) of Fmoc-F3,4-Phe (blue and grey) and Fmoc-F3,5-Phe (orange and yellow). The plotted values are average percent exposures of the Fmoc moiety or Phe sidechain for all detected clusters of a given cluster size (number of building block-monomers per cluster). The error bars correspond to the standard error.

Table S1 Crystallographic data of Fmoc-3,4F-Phe.

**Complex**	**Fmoc-3,4F-Phe**
**CCDC Deposition #**	2043733
**Formula**	C_{26} H_{25} F2 N O_5 S
**Crystal description**	colourless needle
**Crystal size, [mm^3]**	0.2 x 0.01 x 0.01
**FW, [g.mol^{-1}]**	501.53
**Space group**	$P2_12_12_1$
**Crystal system**	Orthorhombic
**a, [Å]**<br>**b, [Å]**<br>**c, [Å]**<br>**α, [°]**<br>**β, [°]**<br>**γ, [°]**	4.9826(1)<br>13.0717(2)<br>36.5189(5)<br>90<br>90<br>90
**Cell volume, [Å^3]**	2378.51(7)
**Z**	4
**ρ_{cacld}, [g.cm^{-3}]**	1.401
**μ, [mm^{-1}]**	1.684
**No. of reflections**	11429
**No. of unique reflections**	4854
**$2\Theta_{max}$, [°]; completeness %**	79.703; 97.7
**R_{int}**	0.0319
**No. of parameters (restraints)**	342 (20)
**Final R^a , wR2**	0.0550, 0.1517
**Final R^b, wR2**	0.0568, 0.1533
**GooF**	1.057

[a] for data with $I > 2\sigma(I)$. [b] for all data.
